# Regulation of Redox Homeostasis by Nonthermal Biocompatible Plasma Discharge in Stem Cell Differentiation

**DOI:** 10.1155/2019/2318680

**Published:** 2019-03-31

**Authors:** Ying Li, Eun Ha Choi, Ihn Han

**Affiliations:** ^1^Department of Plasma-Bio Display, Kwangwoon University, 447-1, Seoul 01897, Republic of Korea; ^2^Plasma Bioscience Research Center, Applied Plasma Medicine Center, Kwangwoon University, 447-1, Seoul 01897, Republic of Korea; ^3^Department of Electronic and Biological Physics, Kwangwoon University, 447-1, Seoul 01897, Republic of Korea

## Abstract

Recently, a growing body of evidence has shown the role of reactive species as secondary messengers in cell proliferation and differentiation, as opposed to the harmful metabolism byproducts that they were previously solely recognized as. Thus, the balance of intracellular reduction-oxidation (redox) homeostasis plays a vital role in the regulation of stem cell self-renewal and differentiation. Nonthermal biocompatible plasma (NBP) has emerged as a novel tool in biomedical applications. Recently, NBP has also emerged as a powerful tool in the tissue engineering field for the surface modification of biomaterial and the promotion of stem cell differentiation by the regulation of intracellular redox biology. NBP can generate various kinds of reactive oxygen species (ROS) and reactive nitrogen species (RNS), which may play the role of the second passenger in the cell signaling network and active antioxidant system in cells. Herein, we review the current knowledge on mechanisms by which NBP regulates cell proliferation and differentiation through redox modification. Considering the importance of redox homeostasis in the regulation of stem cell differentiation, understanding the underlying molecular mechanisms involved will provide important new insights into NBP-induced stem cell differentiation for tissue engineering.

## 1. Redox Homeostasis in Stem Cell Differentiation

The focus of tissue engineering is regenerating damaged tissues through the restoration, maintenance, and improvement of tissue function [1]. For example, in bone tissue, the critical size of bone defects, which lies beyond the spontaneous regeneration capacity of a patient and thus requires surgical invention, has guided research into bone tissue engineering-based therapeutics [2]. Stem cells are the critical cell sources in tissue engineering that possess the characteristics of self-renewal and potential to differentiate into multiple cell types for the repair and/or regeneration of defective tissues and organs, such as the bone, cartilage, heart, neurons, and spinal cord [3–7]. To induce stem cell differentiation, growth factors are the most commonly used technique. Other techniques are also being studied, such as the electromagnetic field, vibration, radiation, heat shock, and oxidative stress [8–17]. Scaffolds provide a framework for stem cells to migrate to, attach to, and specialize on [2]. However, the low efficiency of expansion and differentiation of stem cells *in vitro* is resulting in attempts to develop new methods to improve their characteristics.

Since stem cells are an essential part of tissue regeneration, extensive research has been conducted on the factors regulating stem cell self-renewal and differentiation. Reactive oxygen species (ROS), the highly chemically reactive byproducts of aerobic metabolism, are important mediators in stem cell biology [18, 19]. Changes in ROS levels can be used to monitor the balance of stem cell self-renewal and differentiation. Although high levels of ROS have long been suggested to be detrimental to mediating oxidative stress, mounting experimental evidence indicates that the physiological levels of ROS are involved in the maintenance of intracellular reduction-oxidation (redox) homeostasis and various cellular signaling pathways [20]. ROS in redox homeostasis plays a pivotal role in the maintenance of stem cell self-renewal with low levels of ROS, whereas in differentiated stem cells, ROS is accumulated [21]. For example, a quantitative study comparing human embryonic stem cells (ESC) with their differentiated descendants has shown that ESC are characterized by a lower ROS level, while differentiated cells contain more oxidative species. However, biochemical normalization of the ROS level to cell volume/protein indicates that all cell types maintain a similar intracellular redox of the ROS level as a measure of intracellular redox balance [22]. ROS are also involved in signal transduction cascades in enhancing the differentiation of ESC toward the cardiomyogenic and vascular cell lineage [23]. These findings imply that redox signaling plays a crucial role in modulating the fate of stem cells. Therefore, it is possible that manipulating the exogenous “ROS donor” tool could activate intracellular redox-dependent signaling to maintain stem cell differentiation.

## 2. Nonthermal Biocompatible Plasma (NBP)

Nonthermal biocompatible plasma (NBP) (or plasma) is produced by applying a sufficiently high-voltage electric field across the discharge gap to initiate a breakdown of gas at atmospheric pressure [24]. When NBP is generated, the major components of charged particles, neutral gas species, reactive species, electric field, and radiation are produced. NBP was first employed in antimicrobial applications, because it produces a variety of biotoxic agents that include reactive species, UV radiation, and charged particles. Since then, NBP has come to be extensively studied in other applications in the biomedical field, including in sterilization, cancer cell apoptosis, wound healing, blood coagulation, and teeth whitening [25–31], which has made NBP a promising tool for biomedical use. An increasing number of studies have shown the role of NBP in tissue engineering on the surface modification of biomaterials [32–34] and as an exogenous stimulator that directly induces stem cell proliferation and differentiation [35–40]. In this section, NBP devices and their characteristics will be summarized and analyzed so as to provide a more detailed concept of NBP production and composition.

### 2.1. Classification of NBP Devices

**Figure 1:**
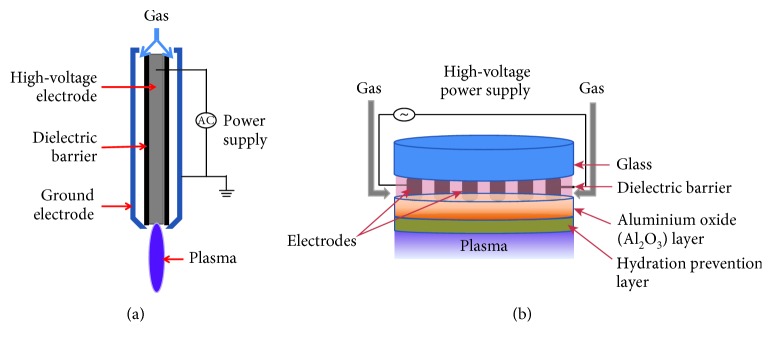
Schematic of the (a) jet type and (b) DBD type of plasma devices to produce NBP.

NBP devices for stem cell differentiation can be broadly classified into two major categories: plasma jet and dielectric barrier discharge (DBD) plasma.  Figure 1 shows an example schematic of a plasma jet and DBD device produced in our research center. The basic structure of the plasma jet type consists of an inner high-voltage electrode, which is coupled with the power source and covered with a dielectric barrier and a grounded outer electrode. Then, either a mixture of noble gas with reactive gas or just pure gas is fed into the annular space between the two electrodes. The plasma ionization degree in the jet is very low (at 10^−6^–10^−7^) (gas density is at 1 atmospheric pressure and 300 K is around 2 × 10^19^ cm^−3^). In the DBD plasma device, the high-voltage and grounded electrodes with an electrode gap of around 400 *μ*m are coated on glass and covered with a dielectric barrier with a thickness of 50 *μ*m. Normally, high-voltage current is alternated between the two electrodes in order to prevent high temperature buildup and transition to arc discharge [41].

### 2.2. RONS Generated by NBP

Several researchers have applied NBP to stem cell differentiation. However, the devices that have been used to generate NBP in these studies have various characteristics in each research group, with the generation of different RONS concentration. Typically, optical emission spectroscopy (OES) is used to show the excited elements in NBP generation, due to the unique emission spectra specific to each element as it transitions from the excited to group state [42]. One of the main factors affecting the NBP-generated RONS species is feeding gas. According to feeding gas, the inert gases, such as argon (Ar) and helium (He), are initially used, because they have relatively lower breakdown voltages. The mixture of inert gas with different percentages of oxygen (O_2_) can generate more ROS [39, 43]. Recently, reactive pure gas, such as nitrogen (N_2_) and air, has come to be mostly used to generate various ROS and RNS. Particularly, when using N_2_ gas, OES shows an RNS-dominant signal, including N_2_ second positive species (SPS), N_2_^∗^, and nitric oxide (NO*_γ_*) (Table 1).

### 2.3. Interaction of NBP with Living Cells

**Figure 2:**
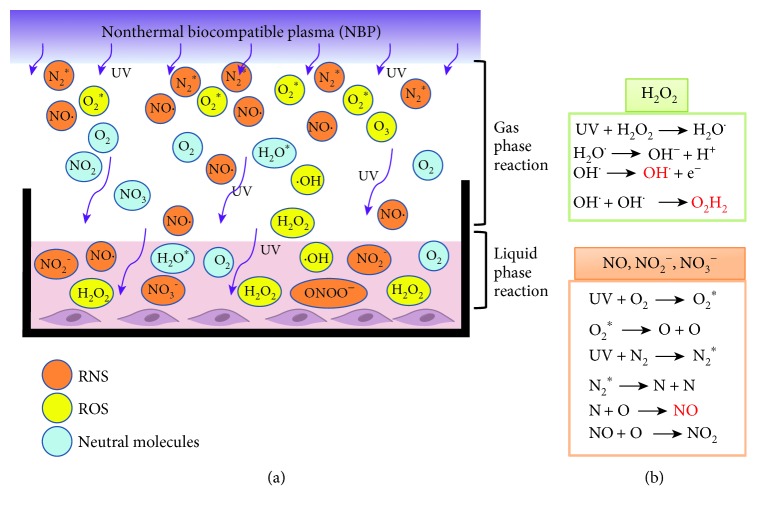
Schematic of NBP-generated ROS and RNS reaction and transportation. (a) NBP-generated radical in the gas phase and liquid phase. (b) Generation of H_2_O_2_ and NO_x_ in the liquid phase by UV photolysis.

Initially, NBP was generated by ionizing neutral gases with high voltage. As shown in Figure 2(a), the surface of culture media was then bombarded by RONS generated during NBP discharge and the reaction of species could be explained by plasma-initiated ultraviolet (UV) photolysis (Figure 2(b)). Then, according to UV absorption spectroscopy, the density of ^•^OH radical inside the solution (2 mm below the solution surface) was higher than that on the surface of the solution. In addition, the lifetime and falling time of ^•^OH radical inside the solution were longer than those above the solution, which could be explained by the following reactions: UV + H_2_O^∗^ →  ^•^OH and UV + H_2_O_2_^∗^ →  ^•^OH inside the solution, as compared with the air environment [42, 44]. The NBP-generated RONS at the cell/environment interface initiates an immediate intracellular oxidative response. These immediate increases of extracellular and intracellular RONS after NBP treatment are mainly due to extrageneous production by plasma, but the extended detection (one hour after treatment [35]) indicates that intracellular RONS are being actively produced in response to NBP, since RONS scavenger could significantly reduce the intracellular RONS level. The most likely sources of the increased intracellular RONS (such as O_2_^•-^, ^•^OH, H_2_O_2_, and NO) are mitochondrial electron transport chain (Mito ETC) and nicotinamide adenine dinucleotide phosphate (NADPH) oxidase [19]. These molecules are signaling factors that are known to participate in cellular proliferation and differentiation either directly, through the activation of ROS or RNS-responsive proteins, or indirectly, through the alteration of the redox status of the cell [35].

**Figure 3:**
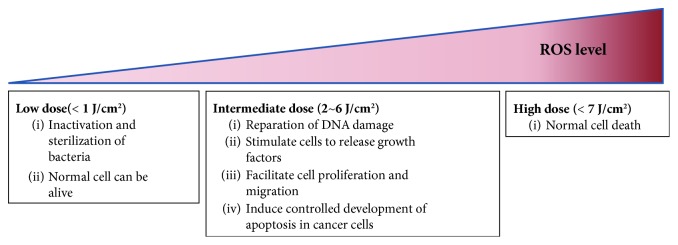
Different effects of NBP interaction with living cells according to NBP dose levels.

NBP interacting with living cells presents a dose-dependent effect; in other words, the NBP doses received by cells could result in various biological reactions.  Figure 3 showed the dose-dependent manner of living cells under NBP treatment. Under the condition of low-dose (<1 J/cm^2^) inactivation and sterilization of bacteria, normal cells can survive. Intermediate doses ((2–6) J/cm^2^) will cause repairable DNA damage, stimulate cells to release cell growth factors, facilitate cell proliferation and migration, and induce controlled development of apoptosis in cancer cells. Under high dose (>7 J/cm^2^) of NBP, normal cell death will happen, and if the NBP doses exceed 10 J/cm^2^, the cell will enter necrosis [45]. The different doses can be controlled by discharge time, power supply, discharge frequency, and even the gas type. In fact, different doses of NBP applied to organic materials will present different effects, such as killing cancer cells or inducing stem cell differentiation. Note that the results presented in this review are generally dependent on the types of plasma devices and stem cell types used in each experiment. Thus, it is important to pay attention to the details of the particular NBP source and stem cell-derived source used in each experiment.

## 3. Effects of NBP on Stem Cell Survival, Proliferation, and Differentiation

### 3.1. Effects of NBP on Stem Cell Survival and Proliferation

It is important to note that, in order to use NBP discharge as a controllable tool for RONS generation, the safe range of NBP doses must be tested before experimentation. Cell viability is a powerful and convenient method for screening of the NBP safe dose range. Indeed, if NBP is going to be considered for potential application in stem cells, the noncytotoxic activity of NBP is a crucial topic.

Osteoprogenitor cell line MLO-A5 was treated with a DBD-type plasma device for various frequencies (at 5, 1000, and 3500 Hz). Treatment of 3500 Hz led to large areas of cell detachment and damage condition, while 1000 Hz treatment increased lactate dehydrogenase (LDH) release after 24 h. Further, Histone 2A variant (H2AX) and cytoplasmic cytochrome c were induced by 1000 Hz for 30 s (12 J/cm^2^) and 60 s (24 J/cm^2^) treatment time, whereas the 1000 Hz for 10 s (4 J/cm^2^) dose did not induce DNA or mitochondrial damage [35]. Similarly, the MC3T3-E1 cell line was treated by the NO plasma jet-type system, with different times (30, 120, and 180 s). There was no significant presence of dead cells, even with prolonged NO-NBP treatment durations of up to 180 s, as compared to control cells. Even when increasing the treatment time (to 360 and 480 s), only 2 and 4%, respectively, cell toxicity was observed [36]. Very recently, in a previous study of ours, we found that the viability of MC3T3-E1 cells treated with a DBD plasma device showed a NBP treatment time-dependent decrease [46].

Aside from progenitor cell lines, the effect of NBP on primary mesenchymal stem cell derived from human periodontal ligament stem cells (hPDLSCs) was studied as well. NBP showed no significant toxicity to cells, even when treated with up to 1.6 W for 120 s with 1 slm helium gas flow [37]. The use of a helium-based DBD device increased the proliferation of adipose tissue-derived stem cells (ASCs), and the cells still maintained their stemness and capability to differentiate into adipocytes; however, no cellular senescence was observed. The proliferation-enhancing ability of NBP treatment was significantly decreased when NO scavenger was added, while no significant change was found with the presence of the ROS scavenger. These results suggest that NBP-generated NO, rather than ROS, is responsible for the enhanced proliferation of ASCs [47]. A recent study indicated that a proper dose of NBP treatment could facilitate neural stem cell differentiation into neurons, which was regulated by the NBP-induced NO production [39]. Low NO concentration was shown to promote cell survival and proliferation in various cells, including stem cells [47], while high NO concentration was shown to lead to cell cycle arrest and cell death [48]. More recently, NBP was found to exhibit a protective role over the neuroblastoma-derived cell line under oxidative stress, suggesting the therapeutic potential of NBP as a novel “NO donor” in neuroprotection and neurodegenerative diseases [49].

### 3.2. Effects of NBP on Stem Cell Migration

Cell migration is central to many biological and pathological processes, including embryogenesis, tissue repair, and regeneration, as well as cancer and the inflammatory response. Research has addressed how low-intensity helium NBP affects cells, such as fibroblasts and endothelial and smooth muscle cells, by inducing the disruption of cell-to-cell adhesion, as well as subsequent cell detachment from the substrates, acting in a dose-dependent manner [50, 51]. More importantly, the observed effects were reversible, since after NBP treatment, the detached cells remained viable and reattached to the plate surface and then, after a short incubation time, continued proliferating. The adhesion assay data showed that treatment with NBP did not significantly affect hPDLSC adhesion, although some detachment of the cells was observed when the higher powers of plasma were combined with 0.5 lpm flow plasma treatment, which considerably decreased the migration capacity of the hPDLSCs. In the study on NBP interaction with cancer cells, invasion and adhesion were suppressed by either NBP directly or NBP-activated media at a certain dilution ratio. In addition, NBP treatment inhibited the matrix metallopeptidase 9 (MMP9) enzyme, which is involved in the degradation of the extracellular matrix during cancer cell migration [52, 53].

### 3.3. Effects of NBP on Stem Cell Differentiation

Osteogenesis is the process consisting of the formation and development of the bone. This process begins at the eighth week of embryo development of bone formation and happens continuously with bone growth until early adulthood; in addition, all of the developments of bone thickness, remodeling, and repairing are connected with osteogenesis. During this process, the osteogenic genes, including alkaline phosphate (ALP), type I collagen (COL-1), Runx-related transcription factor 2 (Runx 2), osteocalcin (OCN), osteopontin (OPN), and osterix, are expressed. These genes are related to bone mineralization and calcium ion homeostasis during the differentiation of stem cells into osteoblasts. One research effort initially suggested that NBP does not significantly promote osteogenesis; however, when inducing stem cells were entering the differentiation stage, NBP could promote more differentiation-specific protein expression [35]. In terms of ALP enzyme activity, it has been shown that the NBP-treated group was significantly higher than the only helium-treated or nontreated group [37]. Later, the NBP effect was studied without differentiation media. Since *ALP* and *COL-1* are early expression genes of osteogenesis, *OPN* and *OCN* are expressed in late differentiation and mineralization, which indicated that NBP could induce early osteogenic differentiation of progenitor/stem cells [36].

In order to determine whether the differentiation effect of NBP was osteoblast specific, the N1511 chondrocyte cell line was treated by NBP with the presence of bone morphogenetic protein 2 (BMP2), a known inducer of chondrocyte differentiation [54]. Twenty-four hours after treatment, chondrocyte differentiation markers *Runx2* and *ALP* were increased 3- to 6-fold above BMP2-treated controls. By 56 hours after NBP treatment, collagen type X (*Col X*) and another late marker, matrix metalloprotease 13 (MMP13), were increased (20- and 4-fold, respectively) above BMP-treated control. The results show that once the chondrogenic differentiation is started, NBP works as a synergic function to the N1511 chondrocyte cell line [35].

Neuronal cells have attracted substantial interest for the medical treatment of neurodegenerative diseases and traumatic injuries of the central nervous system (CNS), but efforts to produce these cells have thus far only been met with modest success. In an attempt to find new approaches, Xiong and his colleague [39] treated neural stem cells (NSCs) with NBP to differentiate them into the neuronal lineage. NBP-treated cells exhibited rapid proliferation and differentiation with longer neurites and cell bodies, eventually forming neuronal networks. The treated cells showed increased expression of different cell lineage markers such as *β-tubulin III* (for neurons) and oligodendrocyte marker, *O4*, while the expression of glial fibrillary acidic protein (*GFAP*) (for astrocytes) remained unchanged.

Taken together, there are several advantages to NBP-induced stem cell differentiation. First, the differentiation process with NBP treatment is faster. Second, the differentiation efficiency dramatically increased with upregulated-specific genes and differentiation signs by NBP treatment. Finally, NBP treatment with/without other chemical inducers could reach a higher percentage of differentiated cells [55]. *In vivo* research has also indicated that NBP promotes neural differentiation into mature neurons in transgenic zebrafish. Specifically, GFP+ mature neurons in developing zebrafish were observed in the central nervous system after 6 h with 1 min NBP treatment and these were maintained through 33 h [55].

## 4. Potential Mechanism of NBP Interaction with Living Cells

### 4.1. NBP Facilitates Intracellular RONS Accumulation and Alters the Antioxidant System

Mechanistically, research has directly linked NBP interaction with living cells via ROS or RNS generation. During NBP discharge, the working gas is ionized into charged particles and chemical species, which then collide with the molecules present in air (O_2_, N_2_, H_2_O, and CO_2_), resulting in the direct formation of numerous RONS [56, 57]. RONS are small, short-lived reactive molecules that display high chemical reactivity toward multiple proteins involved in signaling pathways that regulate cell function. However, the excessive generation of ROS and imbalance between ROS and antioxidant proteins can cause oxidative stress to cells. For example, H_2_O_2_ plays an important physiological role as an intracellular signaling molecule, regulating a wide variety of biological processes, depending on its intracellular concentration (<100 nM) [58]. In osteoblast progenitors, the continuous production of low levels of H_2_O_2_ stimulates proliferation and also augments their potential to differentiate into mature osteoblasts through the upregulation of *Runx2* and *osterix* [35, 59]. Thus, the proper level of ROS, that is, a physiologically sufficient amount, acts as a secondary signaling messenger for stimulating stem cell proliferation and maintains intracellular redox balance for cell survival.

**Figure 4:**
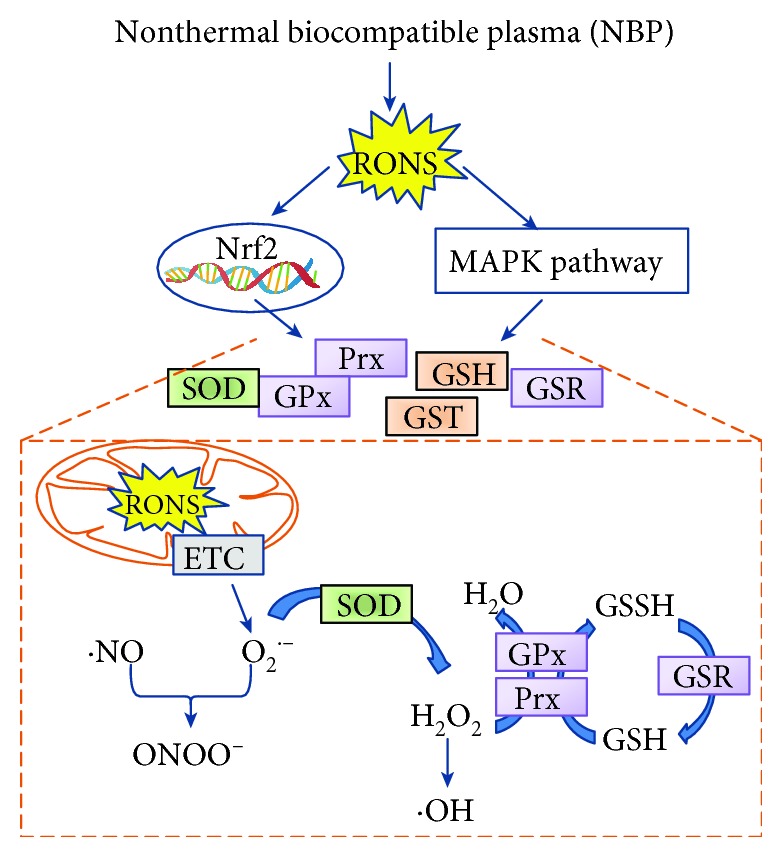
NBP triggers nuclear translocation of nuclear factor erythroid 2-related factor (Nrf2) and mitogen-activated protein kinase (MAPK) signaling pathways to modulate redox homeostasis by altering the antioxidant system and phase II detoxification enzymes and proteins (e.g., superoxide dismutase (SOD), glutathione (GSH), glutathione S-transferase (GST), glutathione reductase (GSR), glutathione peroxidase (GPx), and peroxiredoxin (Prx)).

Table 1 shows a summary of studies that have revealed the extracellular ROS level (in culture media) and intracellular ROS. Based on these, we can observe that the most commonly existing chemical species in culture media following NBP treatment are NO^•^ and H_2_O_2_, while intracellular ROS or RNS accumulation varies from NO to H_2_O_2_ and also includes mitochondria O_2_^•-^. These chemical species are responsible for stem cell lineage commitment to osteoblasts, neurons, myocytes, and chondrocytes. One study reported the role of O_2_^•-^ in neurogenesis: in normal condition, O_2_^•-^ is produced through an electron reduction of oxygen by blocking normal electron transfer in the electron transport chain (ETC). Mitochondria O_2_^•-^ is accumulated via an increase of extracellular NO^•^ concentration, because of NO^•^ competing with oxygen to bind to the active site of the mitochondrial ETC complex IV, cytochrome c oxidase (COX), and reversibly inhibits its activity; therefore, O_2_^•-^ was accumulated in mitochondria [60, 61]. Within the capacity of redox regulation, NBP-generated ROS/RNS trigger redox-sensitive signaling pathways (e.g., nuclear factor erythroid 2-related factor (Nrf2) and mitogen-activated protein kinase (MAPK)) to alter antioxidant enzymes and phase II detoxification proteins, such as superoxide dismutase (SOD), glutathione (GSH), glutathione S-transferase (GST), glutathione reductase (GSR), glutathione peroxidase (GPx), and peroxiredoxin (Prx), to protect cells from oxidative damage [61–63] (as shown in Figure 4).

However, at a high concentration of intracellular ROS levels, which is beyond the capacity for cell self-balance with redox reactions, the cells showed programmed cell death, namely, apoptosis. This phenomenon was observed in various cancer cells treated by NBP (as shown in Table 1). All of these data suggested that the dual role of exogenous RONS or further induction of intracellular RONS levels with killing or stimulation depends on the amount of RONS. More importantly, the redox condition and redox ability of the cells are various, which means that in cancer cells, a high metabolism rate produces relatively high ROS, while stem cells are derived from relatively low ROS concentration niches. Therefore, the effects of NBP treatment to different cell types could vary, even with similar treatment time. However, the ways in which NBP-generated RONS interact at a molecular level in a biological environment, such as cells or cell components, become an open question.

### 4.2. NBP Modification on the Thiol Group of Cysteine Residues

When NBP is applied to biological samples, the most susceptible macromolecules are proteins. RONS bombarded to treatment target diffuses, penetrates the media or body fluid, and reacts with inactivated biological functional biomolecules, such as protein or amino acids. Takai et al. [64] investigated the chemical effects of NBP on 20 naturally occurring amino acids and found that sulfonation and disulfide linkage were formed out of thiol groups in cysteine by NBP treatment. In addition, the formation of aromatic rings by hydroxylation and nitration was found in tyrosines, phenylalanine, and tryptophan, while sulfoxidation was found in methionine and amidation of the ring opening of the five-membrane rings was found in histidine and proline. Another study also reported the NBP modification of phenylalanine by hydroxylation. More recently, the same group confirmed the formation of disulfide linkages between the thiol groups of cysteines by NBP [65, 66], suggesting that redox modifications by NBP-generated RONS of the redox-sensitive cysteine residues are a pivotal mechanism for the functional regulation of a variety of proteins.

**Figure 5:**
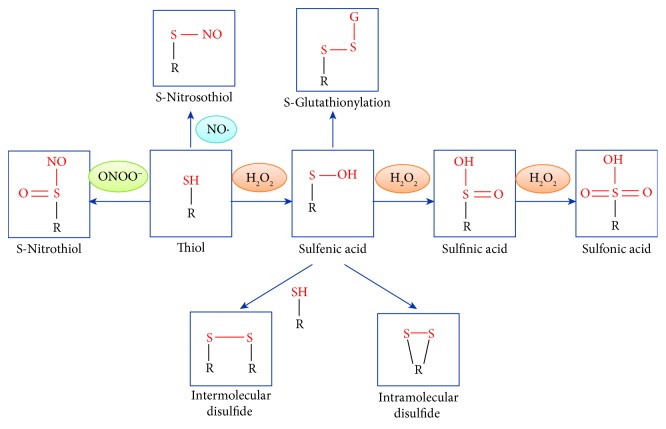
The modification of a redox sensor with a thiol group by NBP-generated RONS.

Cysteine residues that exist as thiolate anions (Cys-S^−^) are more susceptible to oxidation by NBP-generated RONS [67]. In response to oxidation by H_2_O_2_, the thiol group can reversibly form sulfenic acid (Cys-SOH) as well as intramolecular or intermolecular disulfide. The sulfenic form can be reduced to its original state by the disulfide reductases of glutaredoxin (Grx) and thioredoxin (Trx) [68]. However, with the persistent presence of high-concentration H_2_O_2_, sulfenic acid can be irreversibly oxidized into sulfinic acid and, further, into sulfonic acid. For example, the presence of RNS by NO^•^ leads to an S-nitrosothiol bond (SNO), while that by ONOO^−^ leads to an S-nitrothiol group (SNO_2_) [18, 69, 70] (as shown in Figure 5). The reversible reaction between thiol groups and RONS is the way in which NBP interacts with redox-sensitive proteins to further activate or inactivate them.

### 4.3. Regulation of Redox Sensors by NBP-Generated ROS/RNS

**Figure 6:**
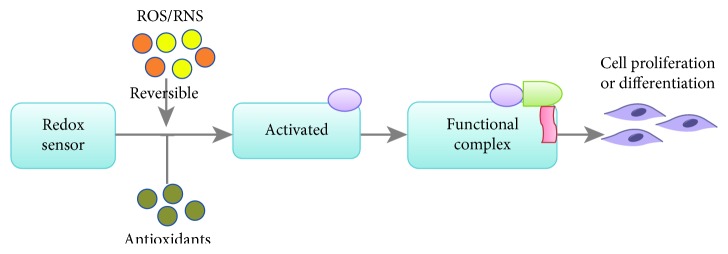
ROS/RNS-mediated intracellular signaling. The physical amount of ROS/RNS can posttranscriptionally modulate the redox sensor protein structure to activate protein and make a functional complex to conduct the downstream signaling pathway. Meanwhile, the antioxidant system in cell can be activated in response to the amount of ROS/RNS in order to maintain intracellular redox homeostasis.

**Figure 7:**
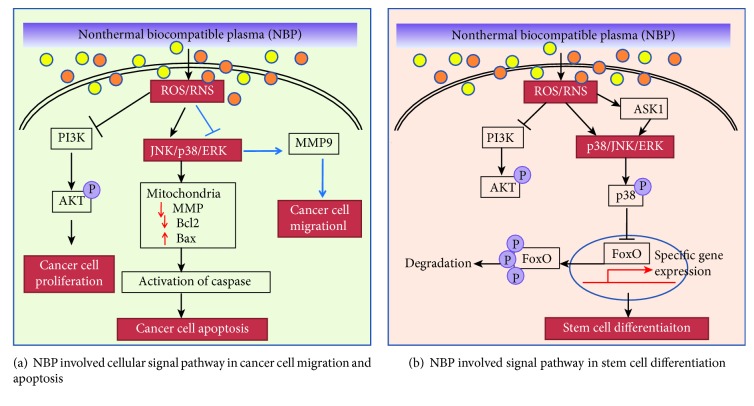
A general summary of the NBP-involved cellular signal pathway in (a) cancer cell proliferation, migration, and apoptosis and in (b) stem cell differentiation.

Most of the key regulators for cell survival and differentiation, including transcriptional factors and kinases, are susceptible to redox modification and recognized as redox sensors [18] (Figure 6). Some of the redox sensors are involved in the regulation of stem cell self-renewal and differentiation, including transcriptional factors and kinases involved in ROS signaling and cell cycle regulation, such as hypoxia-inducible factor-1alpha (HIF-1*α*), forkhead box O (FoxO), p38, c-Jun N-terminal kinases (JNK), phosphoinositide 3-kinase (PI3K), nuclear factor (erythroid-derived 2-) like 2 (Nrf2), and octamer-binding transcription factor 4 (Oct4) [19]. The NBP modifications of these redox sensors are the initial and direct executors of ROS signaling by the activation or inactivation of these proteins. Structural changes during posttranslation modification allow for protein activity to be modified, which can result in altered cellular function [71]. In particular, redox couples are those containing reactive thiol groups, including cysteine, glutathione, and thioredoxin-1. The ROS oxidative modification of these thiol groups initiates signals and promotes downstream cellular responses by affecting the activity and expression of specific transcription factors [72–74]. The cell first perceives extracellular signals and then responds to intracellular ROS through the activation of a variety of signaling pathways, including the mitogen-activated protein kinase (MAPK) and PI3K-Akt pathways.  Figure 7 showed the summary of the NBP-induced signal pathway in cancer cells (Figure 7(a)) and stem cell differentiation (Figure 7(b)).

#### 4.3.1. NBP and MAPK Signaling Pathways

The ROS-responsive MAPK family, consisting of p38, JNK, and extracellular signal-regulated protein kinase (ERK) subfamily factors [75], is known to control a wide range of cellular processes, including cellular differentiation, cell survival, gene expression, cell cycle control, cytokine and growth factor signaling, cell survival, and apoptosis [76, 77]. Survival and proliferation signaling pathways are important cellular signaling circuits which are strongly involved in carcinogenesis.

The MAPK signaling pathway has been well studied in NBP-induced cancer apoptosis. Based on the published literature, JNK and p38 are the most frequently activated MAPKs in response to NBP-induced cancer apoptosis, with effects having been reported in head and neck cancer [78], colorectal cancer [79], thyroid cancer [43], cervical cancer [80], and melanoma [81], among which colorectal cancer cell ERK was also activated. In addition, only the activation of ERK signal was reported in brain and lung cancer cells by H_2_O_2_ and ^•^OH radical-mediated DNA damage [82]. However, adding these different ROS or RNS scavengers, such as ROS scavenger of N-acetylcysteine (NAC), sodium pyruvate, catalase (specific for H_2_O_2_), mannitol and 4-hydroxy-2,2,6,6-tetramethylpiperidinyloxy (specific for O_2_^•-^), and RONS scavenger of butylated hydroxyanisole (BHA), Trolox, and 2-phenyl-4,4,5,5-tetramethylimidazoline-1-oxyl 3-oxide (cPTIO, specific for NO^•^), inhibited the NBP-induced apoptosis of cancer cell, indicating the roles of ROS and RNS in apoptotic signal activation in cancer cells (Table 1).

The MAPKs play various roles in different cell types, and in cancer cell apoptosis, they are mostly activated by NBP treatment; however, in other cell types, MAPKs serve a protective role from apoptosis or even inactivation to inhibit the migration of cancer cells. Bundscherer et al. [83] reported the activation of the MAPK family, including proapoptotic signaling proteins as p38 and JNK and proproliferation signaling protein as ERK in immune cell lines by plasma jet. The main ROS species could be H_2_O_2_, due to the addition of exogenous H_2_O_2_ and plasma treatment having a similar effect. More recently, Li et al. [52] reported the inhibition of cervical cancer cell migration by NBP through the suppression of ERK and JNK of the MAPK family, but not of p38, as well as the downregulation of matrix metalloproteinase- (MMP-) 9 enzyme.

In addition, MAPKs play a vital role in the regulation of stem cell self-renewal and differentiation. The kinase p38*α* as a redox sensor has two cysteine residues, Cys-119 and Cys-162, and was activated by the formation of a disulfide bond [84]; it was therefore considered to be involved in stem cell differentiation. Ito et al. [85] reported that p38 MAPK was activated by increasing the level of ROS. The inhibition of p38 MAPK was also shown to rescue the ROS-induced defects in HSC repopulating capacity and in the maintenance of HSC quiescence, indicating that the ROS-p38 MAPK pathway contributes to exhaustion of the stem cell population and also protects HSCs against loss of self-renewal capacity. Protective roles of p38 MAPK from intracellular oxidative stress have also been found in ESC [86] and NSC [87]. These data indicate that p38 MAPK is a redox regulator that is always activated by ROS in the modulation of stem cell self-renewal and differentiation. Aside from the direct activation of p38 by ROS, the MAP kinase apoptosis signal-regulating kinase 1 (ASK1) is particularly sensitive to ROS, as its activity is tightly regulated by ROS-sensitive proteins, such as thioredoxin and glutaredoxin [77]. ROS-activated ASK1 phosphorylates and activates both p38 and JNK, which play key roles in cellular differentiation [41] as well as the regulation of apoptosis [72]. The activation of ASK1, p38, and/or JNK promotes the differentiation of several cell lineages, including chondrocytes, osteoblasts, neuronal, myoblasts, and keratinocytes [76, 77, 88, 89]. Further, the activation of ERK by its phosphorylation by NBP has been shown 10 min after treatment and this was shown to restore to the normal state at 6 h during NBP-induced neural differentiation through the Ras/ERK signaling pathway [55].

#### 4.3.2. NBP and Phosphatidylinositol 3-Kinase (PI3K)/AKT Pathways

PI3K and its downstream mediator AKT constitute the core components of the PI3K/AKT signaling cascade, regulating cell proliferation, survival, and metabolism [90, 91]. In cancer cells, NBP-generated ROS directly inhibits PI3K/AKT signaling or concurrently activates phosphatase and tensin homolog (PTEN) [92], which negatively regulates the activation of AKT, via oxidizing cysteine residues within the thiol group [93]. The serine/threonine kinase AKT, also known as protein kinase B (PKB), regulates cell survival, death, and cancer development. NBP-induced ROS has been shown to mediate AKT degradation through the activation of the AKT ubiquitin-proteasome system, mitochondrial E3 ubiquitin protein ligase 1 (MUL1) in head and neck cancer cell lines, and therefore to suppress cancer growth *in vitro* and *in vivo* [94]. In stem cell proliferation, NBP treatment significantly reduced PI3K/AKT signaling and MAPK family signaling [46], building the dynamic regulation of cellular proliferation and differentiation, allowing cells to build highly elaborate structures [95].

#### 4.3.3. NBP and FoxO Signaling

Forkhead box O (FoxO) family members are important transcription factors that regulate cellular oxidative stress response by promoting cellular antioxidant defense and thus play important roles in adult stem cells in preserving their proliferative capacity and regenerative potential [96]. The activity of FoxOs in human cells can be directly regulated by the cellular redox state through modifying cysteine residues in FoxOs. ROS induces the formation of the cysteine-thiol disulfide-dependent complex of FoxO on Cys477 and p300/CBP acetyltransferase, and this complex both modulates the biological activity of FoxO-induced cell cycle arrest and enhances FoxO-induced apoptosis [97]. In addition, nucleocytoplasmic shuttling regulates FoxO activity. The results of a recent study suggest that exogenous ROS can activate FoxO through nuclear accumulation by inducing disulfide bridge formation in Cys239 of FoxO4 with transportin-1, which is required for nuclear localization and transcriptional activity [98].

In stem cell differentiation, stimuli such as ROS modulate FoxO activity through phosphorylation and acetylation; moreover, the transcriptional and posttranscriptional gene codings for FoxOs are sensitive to ROS [99]. Yeo et al. [100] reported that FoxO3 coordinates the metabolic pathway by regulating genes in neural stem/progenitor cells (NPCs) for central carbon metabolism of directing the flow of glucose and glutamine carbon into defined metabolic pathways so as to combat excessive ROS, thus maintaining redox balance in NPCs.

On the other hand, FoxO1 is known to be an early molecular regulator during MSC differentiation into osteoblasts. The phosphorylation of FoxO1 leads to its cytoplasmatic retention and the inhibition of its transcriptional activity, while dephosphorylation translocates FoxO1 to the nucleus, where FoxO1 binds to the forkhead response element in the promoter of target genes and interacts with transcriptional coactivators, resulting in the activation of downstream targets. Teixeira et al. [101] revealed that FoxO1 directly interacts with the promoter of Runx2 and coordinates the transcriptional regulation of osteoblast markers. In our previous work, the FoxO1 role in osteogenic differentiation in MC3T3-E1 cells was shown to be related to the phosphorylation of p38. The increase of activated p38 inhibited the phosphorylation of FoxO1, thereby increasing the nuclear accumulation of FoxO1 and transcript osteogenic-related genes [46].

## 5. Conclusion

The groundbreaking studies of NBP for novel regenerative medicine have been expected to lead to a nonlethal oxidative cellular burst that promotes progenitor/stem cell differentiation by the manipulation of intracellular redox homeostasis. Furthermore, the physical levels of ROS and RNS produced in response to NBP influence signaling pathways that are responsible for cellular proliferation and differentiation and mimic the natural intracellular signaling pathway, rather than having deleterious effect. However, it is important to note that there are several advantages and disadvantages of NBP in inducing stem cell differentiation as compared with other strategies, i.e., chemical, biological, and physical stimuli (shown in Table 2). Additionally, further investigation is also needed to clarify the other factors that affect establishing effective differentiation using NBP. Considering NBP can generate controllable amount and mixture ratio of ROS/RNS, which can be benefit to stem cell differentiation process, it would be reasonable to conclude that NBP will play an important role in regenerative therapies with the potential to advance the treatment and management of incurable disease.

## Figures and Tables

**Table 1:** Summary of NBP characteristics, extra- and intracellular ROS/RNS species, and involvement of molecules and signaling pathways in various biological samples of *in vitro* and *in vivo* NBP applications.

Device type	Gas	OES	Extracellular RONS	Intracellular RONS	Scavenger	Cell	Effect	NBP-activated pathway or molecules	NBP-reduced effects	Ref.
Jet	N_2_	NO^•^, N_2_^∗^	NO	NO	cPTIO	Osteoprogenitor cell line	Osteogenesis	*ALP*, *COL-1*	—	[36]
Jet	He	O I, NO^•^, N_2_^∗^, O_3_	—	NO	—	hPDL MSCs	Osteogenesis	ALP enzyme		[37]
Jet	N_2_	NO^•^, N_2_^∗^, O_2_^+^	—	—	—	Animal	Myogenesis	p38, MYH3, MHC, MypG, MyoD	—	[38]
Jet	He + 1%O_2_	^•^OH, O_2_^∗^, O, NO	NO	NO	Hgb	Murine-immortalized neural stem cell line C17.2 Primary rat neural stem cells (isolated from the hippocampus)	Neurogenesis	*β*-Tublin III, APC,	Nestin	[39]
Jet	He	^•^OH, ^•^O_2_^−^	H_2_O_2_	—	—	Osteoprogenitor cells (MC3T3-E1 cell line)	Osteogenesis	ALP enzyme, *ALP*, *OCN*	—	[40]
DBD	Air	—	H_2_O_2_	H_2_O_2_, O_2_^•-^	NAC, TEMPOL	Preosteocytic cell line (MLO-A5); N1511 chondrocyte cell line	Osteogenesis & chondrogenesis	—	—	[35]
DBD	N_2_	NO^•^, N_2_^∗^,^•^OH	H_2_O_2_, NO	—	—	Osteoprogenitor cell line	Osteogenesis	p38, FoxO1	—	[46]
DBD	N_2_ and air	Excited O I	NO	O_2_^•-^ (mitochondrial), H_2_O_2_ (cytosolic)	MitoTEMPO^−^ Trolox, NAC^−^	Mouse neuroblastoma Neuro 2A	Neurogenesis	ERK, Trk, Ras	—	[55]
DBD	He	ROS RNS	—	ROS NO	BHA	Human adipose tissue-derived stem cells (ASCs)	Proliferation	AKT, ERK, NF-*κ*B	—	[47]
Jet	Air	^•^OH, O I, N_2_^∗^	O_2_^−^, ^•^OH, H_2_O_2_	^•^OH, H_2_O_2_	Mannitol, catalase and sodium pyruvate	Brain and lung cancer cell lines	Apoptosis	ERK Bak, bax, caspase,H2AX,	bcl-2	[82]
DBD	He	—	—	—	—	Cervical cancer cell line	Inhibition of migration	—	MMP9 ERK, JNK (cell migration)	[52]
Jet	He + O_2_	^•^OH, O^∗^, O^+^ (~10^13^/m^3^ ROS density)	—	Total ROS	NAC	Head and neck cancer cells	Apoptosis	JNK, p38, caspase 3	Migration invasion	[78]
Jet	He + O_2_	^•^OH, O^∗^, O^+^	—	Total ROS	NAC	Colorectal cancer cell	Apoptosis	p38, JNK, ERK, *β*-catenin	Cyclin D1	[79]
Jet	He + O_2_	O I, NO^•^, O^+^ FNS, N_2_^∗^, ^•^OH	—	Total ROS	NAC	Thyroid cancer cell	Apoptosis	JNK, p38, caspase 3	—	[43]
Jet	Air	H_2_O_2_, O_x_, OH^−^, ^•^O_2_, NO_x_	H_2_O_2_, NO	H_2_O_2_, NO	NAC cPTIO	Cervical cancer cells	Apoptosis	JNK, p38	—	[80]
Jet	He	^•^OH, N_2_^∗^, O I	—	Total ROS, NO	NAC	Melanoma cancer cells	Apoptosis	TNF, ASK-1, JNK, p38 r-H2AX, casp3/7	—	[81]
Jet	Ar	^•^OH, O I, NO, N_2_^∗^	H_2_O_2_	—	NAC	Ovarian cancer cell, human primary mesothelial cells, BALB/C mice	Cancer cell migration	—	MMP9 inhibits JNK and p38	[53]
										
Jet	Ar	—	H_2_O_2_	—	—	Immune cell lines	—	ERK, p38, JNK HSP27 (THP-1)	—	[83]
Jet	Air	—	—	Total ROS	NAC catalase	Breast carcinoma	Apoptosis	PTEN	pAKT, STAT3 pathway, IL-6R pathway	[92]
DBD	Air	^•^OH, O I, N_2_^∗^	—	Total ROS	NAC	Glioblastoma and lung adenocarcinoma cell lines, BALB/c mice	Migration, growth	E-cadherin (epithelial maker)	PI3K/Akt, N-cadherin, Slug, Zeb-1	[102]
Jet	He and O_2_	ROS density with ~10^13^/m^3^	—	Total ROS	NAC	Head and neck cancer cell lines	Growth inhibition	—	Akt degradation	[94]

**Table 2:** Comparison of advantages and disadvantages between NBP and other strategies in inducing stem cell differentiation.

	Advantages	Disadvantages	Reference
Chemical and biological stimuli	High efficiency Easy application Well established High rates of proliferation and differentiation Can be produced in large quantities	High cost Very labile May cause side effects Active multiple signaling pathways and lack of specificity	[103, 104]

Physical stimuli	Manipulation of extracellular and physical environment for cells Proper electric field is benefit for cardiomyogenic differentiation	Heat effect Electrical effect Hard to control the proper amount Standardization required	[105–108]

NBP	Low cost Convenient and portable High availability The main role of exogenous free radical and ROS Easy control of the amount of ROS and RNS Mimic natural biological pathways and have minimum side effects	Mostly research based and very few clinical trials Mechanically not understood well yet Standardization required Low efficiency to differentiate	[35, 36, 46]
